# OTEH-10. Evolutionary trajectory of epigenomic of gliomas

**DOI:** 10.1093/noajnl/vdab070.049

**Published:** 2021-07-05

**Authors:** Tathiane Malta, Thais Sabedot, Indrani Datta, Frederick Varn, AnaValeria Castro, Luciano Garofano, GLASS Consortium, Roel Verhaak, Antonio Iavarone, Laila Poisson, Houtan Noushmehr

**Affiliations:** 1 School of Pharmaceutical Sciences of Ribeirao Preto, University of São Paulo, Ribeirão Preto, São Paulo, Brazil; 2 Department of Neurosurgery, Hermelin Brain Tumor Center, Henry Ford Health System, Detroit, MI, USA; 3 Department of Public Health Sciences, Henry Ford Health System, Detroit, MI, USA; 4 Center for Bioinformatics, Henry Ford Health System, Detroit, MI, USA; 5 The Jackson Laboratory for Genomic Medicine, Farmington, CT, USA; 6 Columbia University Medical Center, New York, NY, USA

## Abstract

Gliomas are the most common malignant brain tumor, have an aggressive behavior, and invariably relapse and progress. Despite the recent advancements, little is known about the role of the epigenome in glioma disease progression and recurrence. To investigate the molecular dynamics over time and in response to therapeutic pressures, the Glioma Longitudinal AnalySiS (GLASS) Consortium, a multinational collaboration, is investigating epigenome-wide molecular data from primary and recurrent matched pairs, including IDH mutant (IDHmut) and IDH wildtype (IDHwt) gliomas. We have compiled a total of 357 samples comprising 143 primary-recurrent pairs profiled by DNA methylation, of which 157 samples have genomic data (WXS/WGS) and 120 have transcriptomic data (RNAseq). IDHwt gliomas have a distinct epigenetic evolution compared to IDHmut after treatment. IDHwt gliomas are more epigenetically stable over time, while IDHmut gliomas display a loss of DNA methylation throughout disease progression. Next, we investigated the molecular drivers of longitudinal gliomas by integration of DNA methylation and gene expression data. We identified epigenetic activation of cell cycle pathways in recurrent IDHmut compared to initial tumors. Transcription factors musculin, ZNF367, and ZNF682 are enriched among recurrent IDHmut gliomas and potentially regulate IDHmut recurrence and/or progression. We next used a DNA methylation-based deconvolution approach to estimate the tumor microenvironment (TME) composition. We found that the TME among IDHmut subtypes (Codel, GCIMP-high, and GCIMP-low) presented less immune infiltration than IDHwt (Classic-like, Mesenchymal-like, and PA-like). Post-treatment, we found a decrease of CD4+T and an increase of CD8+T cells in IDHmut. In conclusion, IDHmut gliomas present a more unstable epigenome, while the epigenome of IDHwt gliomas seems relatively preserved after treatment. We identified potential master regulators of cell cycle deregulation of IDHmut recurrence. Finally, the TME differs across IDHmut and IDHwt gliomas and the cell composition changes over time.

